# Prevalence of intestinal helminth infection among school children in Maksegnit and Enfranz Towns, northwestern Ethiopia, with emphasis on *Schistosoma mansoni* infection

**DOI:** 10.1186/s13071-015-1178-6

**Published:** 2015-10-31

**Authors:** Fikru Gashaw, Mulugeta Aemero, Mengistu Legesse, Beyene Petros, Tilahun Teklehaimanot, Girmay Medhin, Nega Berhe, Yalemtsehay Mekonnen, Berhanu Erko

**Affiliations:** Department of Biology, Kotebe University College, P. O. Box 31248, Addis Ababa, Ethiopia; Department of Biology, College of Natural and Computational Sciences, University of Gondar, P. O. Box 196, Gondar, Ethiopia; Aklilu Lemma Institute of Pathobiology, Addis Ababa University, P.O. Box 1176, Addis Ababa, Ethiopia; Department of Microbial, Cellular and Molecular Biology, College of Natural Sciences, Addis Ababa University, P.O. Box 1176, Addis Ababa, Ethiopia

**Keywords:** *Schistosoma mansoni*, *Biomphalaria pfeifferi*, Maksegnit, Enfranz, Transmission foci, Ethiopia

## Abstract

**Background:**

Schistosomiasis is endemic in Ethiopia and previously unknown transmission foci have been reported from time to time in different parts of the country. Further surveys are required in areas where endemicity of the disease is not known to cover them with control program if transmission is taking place. This study, therefore, aims to assess the magnitude of schistosomiasis mansoni and soil-transmitted helminthiasis in Maksegnit and Enfranz Towns, northwestern Ethiopia.

**Methods:**

Cross-sectional parasitological and malacological surveys were conducted in three schools found in Maksegnit and Enfranz Towns. Stool specimens were collected from 550 randomly selected school children (age range 5 to 17 years) and processed for microscopic examination using Kato-Katz method (single smear per stool sample). Malacological survey was conducted in Gumara and Garno Rivers found in the study areas. *Biomphalaria pfeifferi* snails collected from the two rivers were individually exposed to artificial light in order to induce cercarial shedding. Laboratory-bred Swiss albino mice were exposed to the cercariae and definite identification of the schistosome species was made based on morphology.

**Results:**

The overall prevalence of *S. mansoni* infection was found to be 49 %; however, it varied by schools, with Selam having 60.7 %, and Maksegnit Number 1 and 2 having 45.8 and 39.6 %, respectively. The respective mean intensity of *S. mansoni* infection among school children in Selam, Maksegnit Number 1 and Maksegnit Number 2 Schools were 243, 194 and 183 eggs per gram of stool (epg). In all the study areas there was no difference in prevalence of *S. mansoni* infection in relation to age, however, the prevalence varied by sex, with males having highest prevalence (54.5 % vs 44.1 %) (*p* = 0.012). Adult *S. mansoni* worms were harvested from mice exposed to cercariae shed from *B. pfeifferi* on the 6^th^ week post-exposure. The prevalence of *Ascaris lumbricoides* single infection was 16.5 % while its co-infection with *S. mansoni* was 18.2 %.

**Conclusion:**

Infections of young children, findings of schistosome infected snails, establishment of mice infection and harvesting adult worms from the lab-bred mice confirm that autochthonous transmission is taking place in the study areas. Hence, preventive chemotherapy with praziquantel should be put in place, complemented with other measures such as provision of sanitary facilities and health education, to control morbidity and transmission of schistosomiasis and soil-transmitted helminthiasis in the study areas.

## Background

Schistosomiasis ranks second to malaria among parasitic diseases in terms of human morbidity with significant economic and public health importance. Typically, schistosomiasis is a disease that affects rural communities, particularly those who depend on irrigation for agricultural activities [1]. Of schistosome parasites that infect humans, over 90 % of infections are caused by *Schistosoma mansoni, S. haematobium* and *S. japonicum* [2, 3]. They cause health complications that may lead to death [4] with annual mortality rate exceeding 200,000 [5] among an estimated 200million infected individuals, mainly in Africa [6].

In Ethiopia, both intestinal and urinary types of schistosomiasis occur although the former is the most widespread and highly prevalent. Intestinal schistosomiasis due to *Schistosoma mansoni* is transmitted by *Biomphalaria pfeifferi* and *B. sudanica* whereas urinary schistosomiasis due to *S. haematobium* is transmitted by *Bulinus abyssinicus* and *Bu. africanus* [7]. According to Kloos et al*.* [8], most transmission sites of intestinal schistosomiasis are found in agricultural communities living along streams between 1300 and 2000 m altitude. Kloos and colleagues also described that *S. mansoni* transmission above 2200 m and below 800 m is precluded in many parts of Ethiopia by low and high water temperatures, respectively.

It is estimated that 30 million people are at risk of schistosomiasis in Ethiopia [9]*.* Although recent estimate has not been made on the burden of the disease, periodic surveys have shown that schistosomiasis is on the increase in connection with water resource development and intense population movement [10–12]. Hence, continuous epidemiological surveys are required to discover unknown schistosomiasis transmission foci and treat with the control program.

Soil-transmitted helminths infections, especially ascariasis and trichuriasis are also widespread and rampant in Ethiopia reaching as high as over 90 % in prevalence and with heavy burden of infection especially in children in localities where the sanitary conditions are very poor [13, 14]. It is now well recognized that intestinal parasites, including soil-transmitted helminthiasis and schistosomiasis, adversely affect the physical and mental development of the infected children [15]. The objective of this study was, therefore, to determine the magnitude of schistosomiasis mansoni and soil-transmitted helminthiasis in Maksegnit and Enfranz Towns, of northwestern Ethiopia

## Methods

### The study areas

**Fig. 1:**
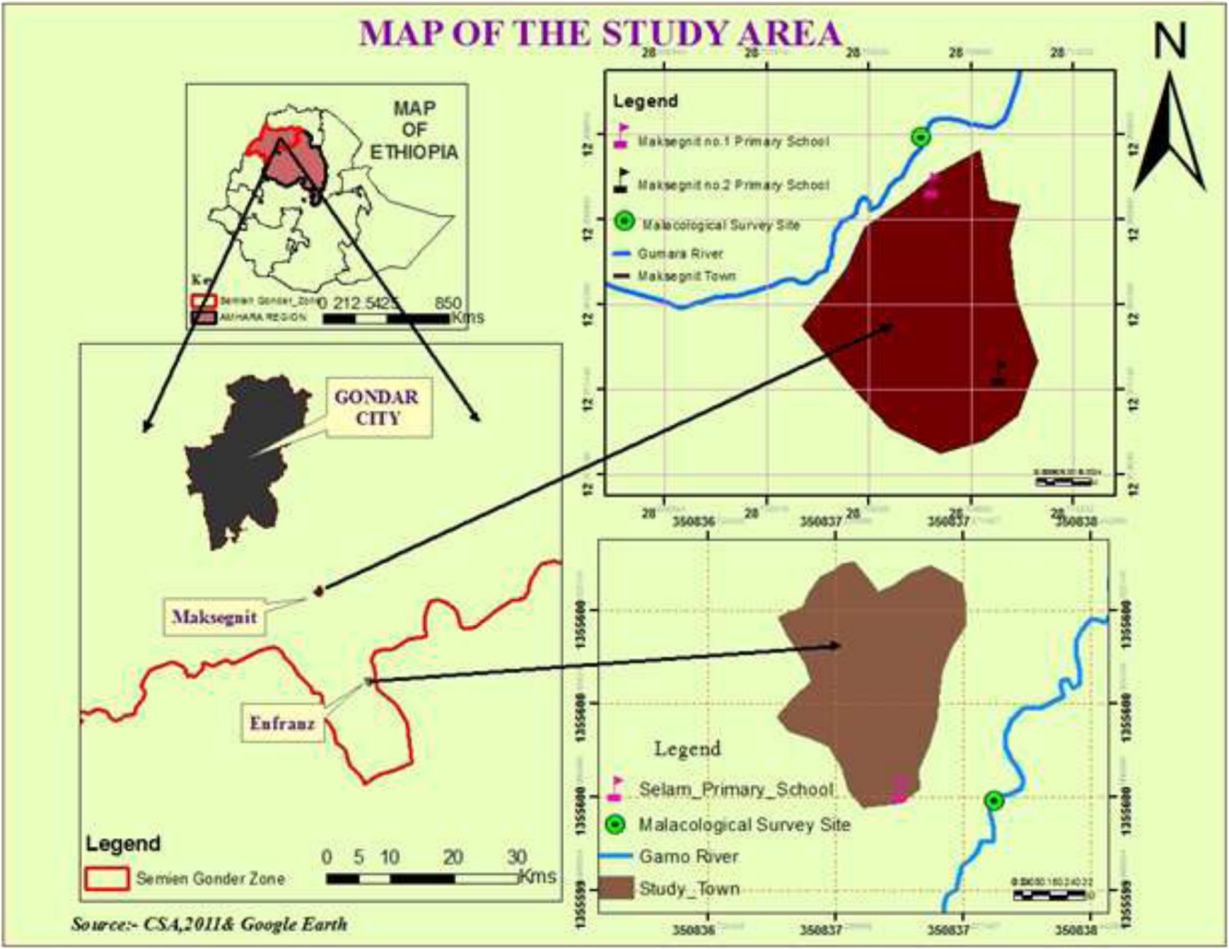
Map of the study area showing the Towns, schools and where snail survey is conducted

Cross sectional parasitological and malacological surveys were conducted in Maksegnit and Enfranz Towns, northwestern Ethiopia, in March 2013 (Fig. 1). The towns are located 42 and 54 km to the south of Gondar City, respectively. In Maksegnit Town, there are two primary schools, namely Maksegnit Number 1 and Number 2 in which parasitological survey was conducted. Malacological survey was also carried out at Gumara River which was close to Maksegnit Number 1 School. The school is located at an altitude of 1940 m above sea level (m.a.s.l.) and lies between 12° 23' 29'' N latitude and 37° 33' 39'' E longitudes. Maksegnit Number 2 School is found at an altitude of 1958 m.a.s.l. and found between 12° 22' 45'' N latitude and 37° 33' 45'' E longitudes.

**Fig. 2:**
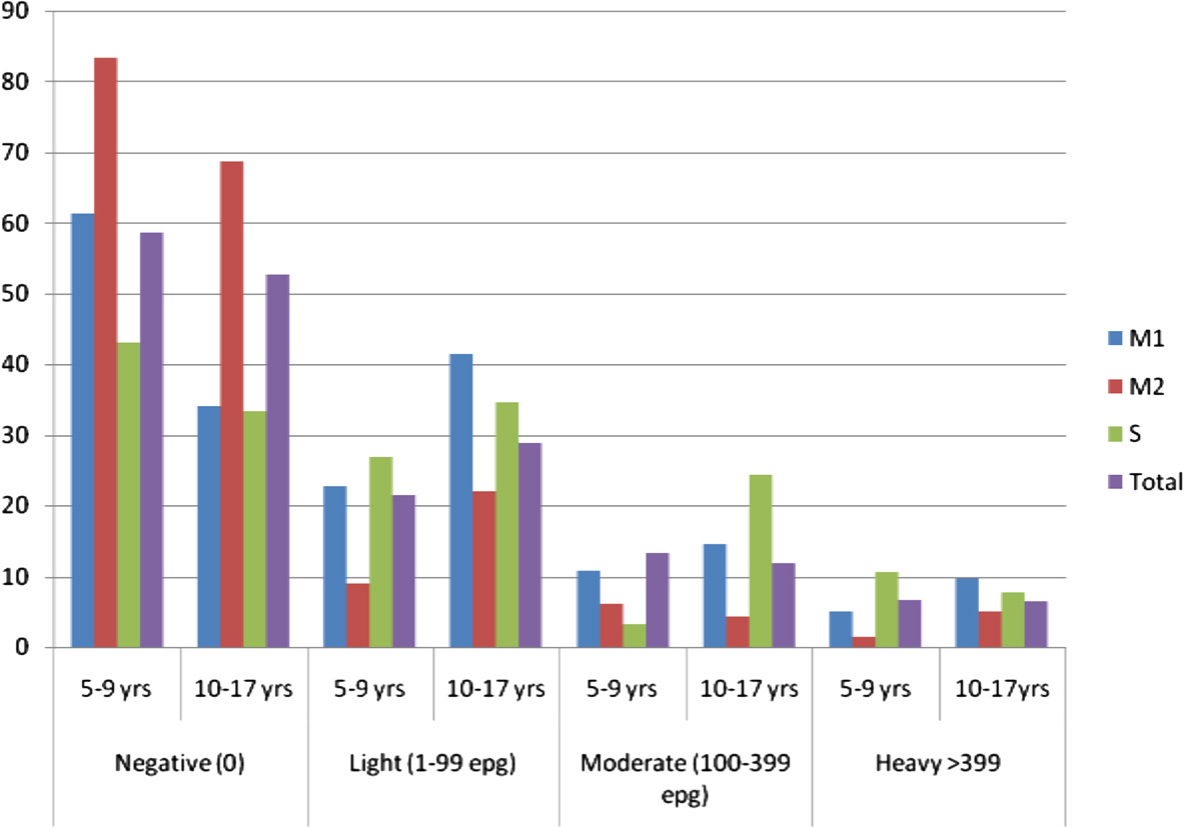
Schistosoma mansoni transmission site and human water contact activities in Gumara (right) and Garno (left) Rivers

In Enfranz Town there are also two primary schools of which Selam Primary School was selected for this study. The selection was based on the presence of a nearby Garno River where there is human-water contact. The school is found at an altitude of 1913 m.a.s.l. and lies between 12° 15' 53'' N latitude and 37° 37' 48'' E longitudes. Both Gumara (from Maksegnit) and Garno (from Enfranz) Rivers are used for washing clothes, bathing, drinking, agricultural irrigation schemes and other domestic purposes. In both of the towns, the major occupants of the inhabitants include civil servants, business men, daily laborers and people using subsistence agriculture especially in the nearby rural areas (Fig. 2).

### Study population and sample selection

School children of Maksegnit Number 1 (Grades 1 and 2), Maksegnit Number 2 (Grade1 to 5) and Selam (Grade 1 to 4) primary schools, attending class during the study period, whose age ranges from 5–17 years *(*mean age being 9.8 years) were the study population. The selection of school children to assess community infection prevalence was based on the reports made by Guyatt et al. [16] Sample size (n) of the study participants was determined using statistical formula *n* = z^2^ p (1-p)/ d^2^ [17] where, *n* = minimum number of sample size, z = statistic for a level of confidence, *p* = expected prevalence, and d = margin of error or confidence interval. Since the overall prevalence rate (p) of intestinal parasites was not known for the study area, we took the p value as 50 %. For the calculation 95 % confidence interval Z was 1.96 and 5 % margin of error (d) was used. This gave the minimum sample required for the study to be 384. In our study, to compensate refusal to participate in the study, minimize errors due to sample collection or Kato-Katz preparation and to have better coverage of the study population with available resources, it was decided to take an additional 166 (30 %) samples. Accordingly, the number of students examined was 142 from Maksegnit Number 1, 207 from Maksegnit Number 2 and 201 from Selam primary schools, totaling 550.

The children were selected by systematic random sampling. First, classes were selected from each school with proportional allocation to size of classes. Then, the study participants were selected using the school registration book as a sampling frame. The first child was selected randomly from the registration book, and then every n^th^ child was selected based on proportional allocation until the required sample was drawn from each class.

### Stool collection and examination

A plastic sheet and applicator stick was given to the selected students to bring sizable stool. Slides were labeled with identification number and the school code. Information with respect to the students name, age, sex, grade, section, length of stay in the study areas were registered corresponding to their code numbers on the spot of sample collection.

Kato-Katz thick smear was prepared by an experienced laboratory technician. Stool microscopy was done using Kato-Katz thick smear method [18]. A single Kato-Katz thick smear per stool sample was prepared from a single stool using a template delivering 41.7 mg of faeces. The slides were transported to Aklilu Lemma Institute of Pathobiology Medical Parasitology lab for microscopic examination. The specimens were examined systematically by the same experienced laboratory technician who prepared the smears. Hookworm egg count was not done as their eggs over-clear after one hour of Kato-Katz smear preparation.

Quality control was assured by using laboratory technicians who have considerable experience in preparing Kato Katz smears and microscopy of stool samples. Study participants were clearly instructed to bring stool samples of their own in the labeled containers provided. The samples were checked for labeling when submitted by the participants. 10 % of the slides were randomly selected and re-examined by a senior laboratory technician who did not previously examine the slides. In case of discordant results, a third senior laboratory technician was asked to examine the slide and readings on which two technicians agreed would be considered.

### Malacological survey

Snail intermediate hosts were surveyed in both Gumara and Garno Rivers in March 2013; a month with occasional showers. The likely habitats such as stones and decaying woods were searched for snails using scoop and by hand picking using gloves and forceps. Collected snails were placed in plastic bucket containing aquatic weeds and transported to Aklilu Lemma Institute of Pathobiology for further investigations. During snail collection, visual observation was made on water contact activities of humans.

Snails were individually placed in shedding vials with aged water and exposed to electric light for about half an hour to induce cercaria shedding. The cercariae were checked by putting the vials under dissecting microscope and identified to the genus level using tail morphology and swimming behavior [19].

Laboratory bred albino mice were exposed to cercariae for about 30 min immersed in beakers containing cercaria infested water. The feces of the mice were checked for the presence of schistosome eggs on the 43^rd^ day. Mice were perfused [20] for harvesting and identification of schistosome worms from blood vessels around the mesentery on the 45^th^ day post-exposure.

### Data analysis

Data were entered into Microsoft Excel data sheet and exported to Statistical Package for Social Sciences (SPSS) software version 16.0 for statistical analysis. The Chi-square test was used to test possible association of infection with age and sex while independent t-test was used to compare mean egg counts by sex and age groups.

A multiplication factor of 24 was used to estimate the number of eggs per gram of stool as the Kato template used delivered 41.7 mg of stool plug. Based on the number of egg counts, the intensity of *S. mansoni* and *A. lumbricoides* infections was categorized as light, moderate, and heavy-infection intensities as per the cutoffs described by the World Health Organization [21]. Thus, intensity of *S. mansoni* was classified as light (1–99 epg), moderate (100–399 epg) and heavy infections (>399 epg). Similarly *A. lumbricoides* infection was classified as light (1- 4999 epg), moderate (5000–49999 epg) and heavy infection (>50,000epg). In the results, only intensities of *S. mansoni* and *A. lumbricoides* were presented since the intensities in all other parasites were light. Prevalence and intensity of *S. mansoni* infection were expressed as percentage and eggs per gram of stool (epg), respectively. *P*-values less than 0.05 are considered statistically significant.

### Ethical consideration

The project obtained ethical clearance from the Institutional Review Board (IRB) of Aklilu Lemma Institute of Pathobiology. Permission to conduct the study was also obtained from both Maksegnit and Enfranz Towns Health Bureaus and school directors. The children also gave their assent before collecting stool specimens. All study participants found positive for intestinal schistosomiasis and other soil transmitted helminths were treated by a health professional with 600 mg praziquantel and 400 mg albendazole, respectively.

## Results

All schools combined, a total of 550 children were examined for helminths infections and 66.4 % were found positive for any intestinal helminths infections. The prevalence of *S. mansoni* infection among school children in Maksegnit Number 1, Maksegnit Number 2 in Maksegnit Town and Selam Primary School in Enfranz Town were 45.6, 39.6 and 60.7 %, respectively. In all the study areas there was no age difference in prevalence of *S. mansoni* infection, however, the prevalence varied by sex, with males having the higher prevalence (54.5 % vs 44.1 %) (*p* = 0.012).

**Fig. 3:**
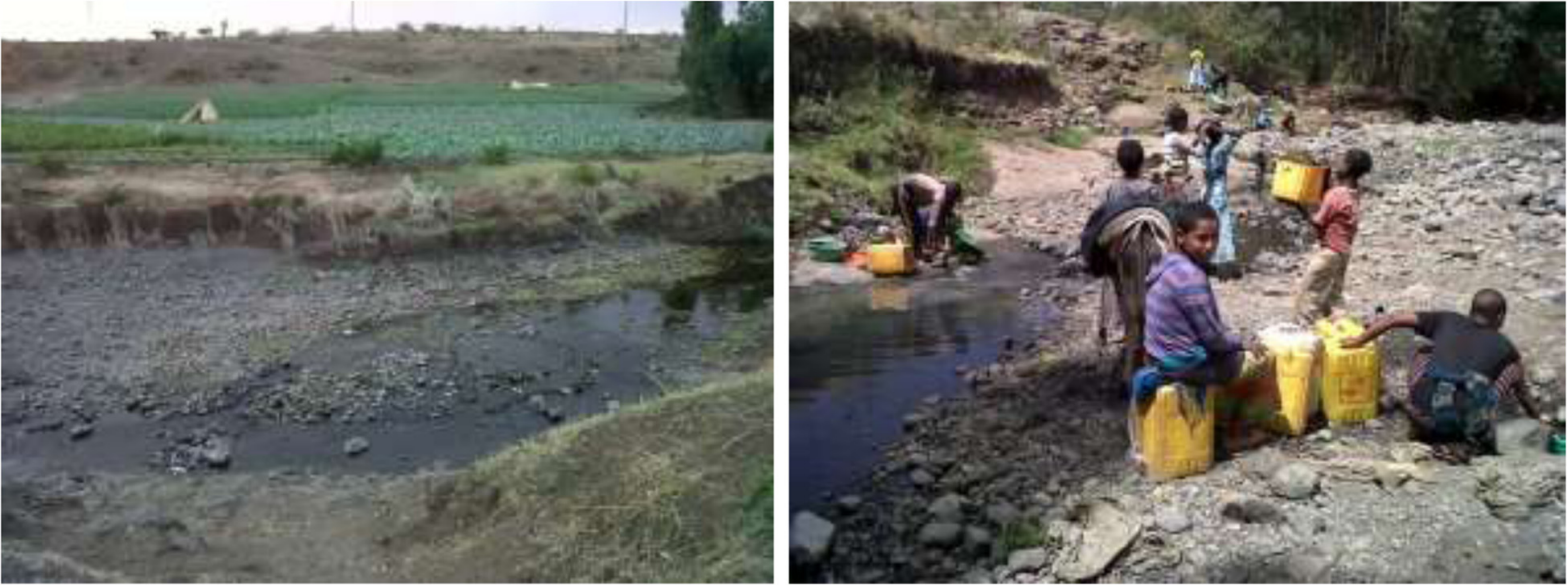
Comparison of *Schistosoma mansoni* intensity of infection in relation to age categories within study subjects among schools

The mean intensity of *S. mansoni* infection among school children in Maksegnit Number 1 was 194 epg (egg count ranging from 24 to 2616 epg) while in Maksegnit Number 2 the mean intensity was183 epg (egg count ranging from 24 to 720 epg). The mean intensity of *S. mansoni* infection among Selam Primary School children in Enfranz Town was 243 epg (egg count ranging from 24 to 2544 epg). The intensity of *Schistosoma mansoni* infection in all of the three schools was higher among males than females (*P* = 0.012). Though the intensity of infection was higher among males than females it was observed that light infection was more common than moderate and heavy infections in all of the three study schools (Fig. 3).

In relation to age the highest intensity of infection was observed among the 5–9 years of age group in Maksegnit Number 1 School and in the 10–17 years of age group among children in Maksegnit Number 2 School. In Selam Primary School, the highest intensity of infection was observed among 10–17 years age group males and 5–9 years age group females. However, intensity of *S. mansoni* infection didn’t differ significantly by age group (*p* > 0.05) (Table 1).

**Table 1:** Prevalence and intensity of *Schistosoma mansoni* infections among school children in Maksegnit and Enfranz Towns, northwest Ethiopia, March 2013

School	Age group (years)	No. examined (% infected, epg)		
		Male	Female	Total
Maksegnit N^⍛^1	5 - 9	46 (54.3, 260)	55 (23.6, 137)	101 (37.6, 218)
	10 – 17	21 (66.7, 193)	20 (65, 124)	41 (65.9, 160)
	Total	67 (58.2, 236)	75 (34.7, 130)	142 (45.6, 194)
Maksegnit N^⍛^2	5 - 9	23 (43.5, 149)	43 (51.2, 116)	66 (48.9, 126)
	10 – 17	60 (38.3, 243)	81 (33.3, 202)	141 (35.5, 221)
	Total	83 (39.8, 215)	124 (39.5, 163)	207 (39.6, 183)
Selam	5 - 9	63 (61.9, 261)	60 (51.7, 250)	123 (56.9, 256)
	10 – 17	42 (66.7, 339)	36 (66.7, 95)	78 (66.7, 232)
	Total	105 (63.8, 294)	96 (57.3, 182)	201 (60.7, 243)
All schools combined	5 - 9	132 (56.1, 246)	158 (41.8, 183)	290 (48.3, 216)
	10 – 17	123 (52.8, 274)	137 (46.7, 146)	260 (49.6, 211)
	Total	255 (54.5, 259)	295 (44.1, 165)	550 (48.9, 213)

From the total helminth infected children, 45.6 % had single infection while 20.2 % had double, and 0.4 % had triple infections. *S. mansoni* and *A. lumbricoides* single and co-infection were the most prevalent. The prevalence of *S. mansoni* single infection was the highest (28.7 %) observed in the area. Of the soil-transmitted intestinal helminths, *A. lumbricoides* was more prevalent among Maksegnit Number 2 Primary School children and less among Selam Primary School children.

The total prevalence of *Ascaris lumbricoides* among the school children of the three schools ranged from 10,9 to 51.4 % (Table 2). The intensity of *A. lumbricoides* infection ranged from 24 to 14,760 epg. Among 194 (35.3 %) school children infected with *A. lumbricoides*, 149 (76.8 %) had light infection with mean intensity of 549 epg and 45 (23.2 %) had moderate infection with mean intensity of 7961 epg. Heavy intensity of *A. lumbricoides* infection was not observed.

**Table 2:** Prevalence of *A. lumbricoides* infection among school children in Maksegnit and Enfranz Towns, northwest Ethiopia, March 2013

School	% Positive (No. Examined)	% Positive (No. Examined)	% Positive (No. Examined)
	Male	Female	Total
Maksegnit No.1	47.8 (67)	54.7 (75)	51.4 (142)
Maksegnit No. 2	41.0 (83)	51.6 (124)	47.3 (207)
Selam	15.1 (106)	6.3 (95)	10.9 (201)
Total	32.0 (256)	37.8 (294)	35.1 (550)

The status of hookworm infection was not determined in the study sites since the slides were not examined within one hour of Kato smear preparation. Other rare helminth infections found in the study area were *Taenia saginata*, *Trichuris trichiura* and *Enterobius vermicularis*.

### Malacological study

A total of 61 snails from Gumara and 75 snails from Garno River were collected. Their sizes were measured using caliper. The size of snails from Gumara River ranged from 6 to 13.5 mm in diameter. Similarly, those snails collected from Garno River ranged from 5 to 13 mm in diameter.

Snails collected from both rivers were identified as *B. pfeifferi.* Snails collected from Gumara River shed schistosome and echinostome cercariae. However, snails from Garno River in Enfranz shed only echinostome cercariae. Since parasitological findings of the nearby Selam Primary School showed over 60 % of infection with *S. mansoni*, 14 snails were collected for the second time of which 14.3 % of them shed a number of schistosome cercariae.

Faeces of lab-bred mice exposed to *S. mansoni* cercariae were checked for ova and found negative on the 43^rd^ day post exposure. However, the mice were sacrificed on the 45^th^ day and 88 *S. mansoni* adult male worms were harvested from a single mouse infected with cercariae shed from *B. pfeifferi* collected from Gumara River, as well as 2 adult male worms from the mouse exposed to cercaria from *B. pfeifferi* collected from Garno River.

Visual observation of river habitat characteristics made during snail survey showed that the flow of the river was slow, the water was slightly turbid, vegetation was abundant (both emergent and submerged) and there was also algal growth in the rivers.

## Discussion

The overall prevalence of *S. mansoni* infection in the present study areas was higher (49 %) than the prevalence previously reported from different parts of Ethiopia, which ranged from 0.1 to 38 % [22–24]. In the present study there was also a difference in prevalence of *S. mansoni* infection among school children in Maksegnit Number 1 (45.8 %), Maksegnit Number 2 (39.6 %) and Selam (60.7 %) Primary Schools. The difference in prevalence between the schools in our present study and the previous reports could in part be explained by intensity of parasite transmission, proximity of transmission sites to schools, and frequency of exposure to cercariae-contaminated water bodies.

High rate of *S. mansoni* infection among males (54.5 %) than females (44.1 %) in the present study agrees with reports of earlier studies in different parts of the country [25–30]. This difference in infection rate could be associated with swimming behavior and working on irrigated agricultural farm lands; activities which are mainly performed by males. On the other hand, some other studies reported absence of differences in *S. mansoni* infection between male and female children [31–33]; this can also be explained by similar exposure of male and female children to water contaminated with schistosome cercariae.

There was no significant difference among children in the 5–9 and 10–17 year age groups in infection rate. This could be due to equal exposure of children in these age groups to water contaminated with schistosome cercariae. Some previous studies from different parts of the country also reported similar patterns of infection for these age groups [34, 35].

The communities in Maksegnit Town represent moderate risk community for schistosomiasis transmission according to WHO guidelines [36] as the prevalence rate is between 10–49 %. In this case, it is recommended to treat all school-age children (enrolled and not enrolled) once every 2 years as well as adults considered to be at risk. On the other hand, the overall prevalence of schistosomiasis among children in Selam Primary School was 60.08 %. Hence, communities in Enfranz Town represent high risk (prevalence ≥ 50 %) and the treatment strategy is to treat all school-age children (enrolled and not enrolled) once a year as well as adults considered to be at risk.

*B. pfeifferi* collected from the Gumara and Garno Rivers shed schistosome cercariae which were later definitely identified as *S. mansoni* by establishing the life cycle in the lab-bred mice. The occurrence of schistosome infected *B. pfeifferi* and high prevalence of *S. mansoni* infection among young school children show autochthonous transmission of *S. mansoni* in the study areas. Nevertheless, it is difficult to say whether the transmission is of recent establishment or had been there from time immemorial because of a lack of baseline information for the study areas.

Visual observation of river habitat characteristics during snail survey showed that the flow of the river was slow, the water was slightly turbid, vegetation was abundant (both emergent and submerged) and there was also algal growth in the rivers. Such habitat characteristics are suitable for snail fauna. In previous study, Lo et al*.* [37] described that small streams with flow rate of 10–30 cm/s, slight turbidity, the presence of vegetation and muddy substrata potentially favor biomphalarid, bulinid and lymnaeid snails in water bodies in other parts of Ethiopia. Observation made on human water contact activities also showed that swimming, bathing, crossing the river, fetching water and washing in contaminated water, and watering vegetation were found to be the major exposure activities. Furthermore, open defecation in fields and on river banks was contaminative activity observed mainly among children. Hence, both ecological conditions, human water contact as well as exposure activities favour transmission of schistosomiasis in the study areas.

Although the sanitary conditions leave a lot to be desired in the study areas, the prevalence of soil-transmitted helminths infection was generally low. The highest prevalence recorded was 16.9 %, which was for *Ascaris lumbricoides*. The status of hookworm infection in the study areas has not been determined because stool was not examined within one hour of collection. It is known that hookworm eggs are rapidly over cleared when using the Kato–Katz method because of glycerol-soaked cellophane strips [38, 39]. Hence, in tropical climates, hookworm eggs disappear 30–60 min after preparation of Kato Katz smear [40].

Another limitation of this study is examination of a single Kato smear when two Kato smears from a single stool are currently recommended for diagnosing *Schistosoma mansoni* infections because of its low sensitivity at low infection intensities [41]. Had multiple Kato smears been used, the prevalence of infection would have been higher than what was reported by a single Kato smear per stool sample in the present study areas.

## Conclusion

Infections of young children, findings of schistosome infected snails, establishment of mice infection and harvesting adult worms from the lab-bred mice confirm that autochthonous transmission is taking place in the study areas. Hence, preventive chemotherapy with praziquantel should be put in place, complemented with other measures such as provision of sanitary facilities and health education, to control morbidity and transmission of schistosomiasis and soil-transmitted helminthiasis in the study areas.
